# Whole-Genome Sequence of Pseudomonas sp. Strain MM211, Isolated from Soil in Langenfeld, Germany

**DOI:** 10.1128/mra.01048-21

**Published:** 2022-02-03

**Authors:** Donat Wulf, Jonas Arndt, Isabell E. Bleile, Charlotte Bramers, Pia L. Gülpen, Bart Verwaaijen

**Affiliations:** a Computational Biology, Faculty of Biology, Bielefeld University, Bielefeld, Germany; b Graduate School DILS, Bielefeld Institute for Bioinformatics Infrastructure, Bielefeld University, Bielefeld, Germany; c Molecular Biotechnology, Bielefeld University, Bielefeld, Germany; d Biology, Bielefeld University, Bielefeld, Germany; e Molecular Biology, Bielefeld University, Bielefeld, Germany; Loyola University Chicago

## Abstract

Here, we present the genome sequence of Pseudomonas sp. strain MM211, which was isolated from garden soil. The complete circular genome consists of a 5,281,862-bp chromosome, with a GC content of 61.5%.

## ANNOUNCEMENT

The Gram-negative rod-shaped bacterial genus Pseudomonas lives in diverse habitats (1–3) and is well characterized (4). Currently, 258 validated species are published (5), including human, animal, and plant pathogens (6). In addition, some species interact with plants and can promote plant growth and influence resistance against plant diseases (7, 8). Some Pseudomonas species are able to grow in association with other organisms in highly polluted environments and degrade various substances (9). Because of these many different properties, the organisms of this genus have great potential to be some of the most influential bacteria in research and development (10).

We isolated Pseudomonas sp. strain MM211 from a soil sample obtained in Langenfeld, North Rhine-Westphalia, Germany (51°06′31.1″N, 6°56′40.2″E), from dark humus at a depth of 10 cm. The sample was diluted with 0.9 NaCl, filtered (431015; Macherey-Nagel, Düren, Germany), plated (1.5% agar, 1% peptone from soy, 0.3% NaCl, 0.1% sucrose, 0.1% cellulose, 0.1% xylan, 0.1% chitin, and 0.05% Tris-HCl), and incubated at 28°C until colonies were observed. DNA was isolated from a single colony with a NucleoSpin microbial DNA minikit (Macherey-Nagel) with RNA digestion. DNA was barcoded with the native barcoding kit (Oxford Nanopore Technologies, Oxford, UK) and sequenced on a GridION system with a R9.4.1 flow cell (Oxford Nanopore Technologies). Sequences were called using the super accuracy base-calling model in MinKNOW (v1.4.3; Oxford Nanopore Technologies). Adapters were trimmed using Porechop (v0.2.4) (11). The genome was assembled with Canu (v2.1.1) (12) set to a genome size of 8 Mb and was polished with Racon (v1.4.20) (13) in combination with BWA (v0.7.17) (14) and Medaka (v1.4.3; Oxford Nanopore Technologies). Completeness was examined with Benchmarking Universal Single-Copy Orthologs (BUSCO) (v5.1.2) (15) set to genome, with the lineage set to pseudomonadales_odb10. The final single-contig assembly was circularized and oriented with berokka (v0.2.3) (https://github.com/tseemann/berokka) and uploaded to NCBI. Default settings were used for all tools unless stated otherwise. All relevant assembly statistics, including BUSCO results, are listed in Table 1.

**TABLE 1:** Sequencing and assembly statistics for Pseudomonas sp. strain MM211

Parameter^*a*^	Finding
Raw read sequencing	
No. of reads	168,644
* N*_50_ (bp)	13,834
Total length (bp)	1,579,810,087
Assembly	
Coverage (×)	286
GC content (%)	61.5
Length (bp)	5,281,862
Annotation	
Total no. of genes	4,853
No. of coding genes	4,645
BUSCO results (%)	
Complete	98.8
Single copy	98.3
Duplicated	0.5
Fragmented	0.4
Missing	0.8

a Coverage was based on mapping of the trimmed reads to the assembly with SAMtools (v1.12) (25). Annotation was based on NCBI PGAP (v5.3) annotation of GCA_020386635.1 on 15 November 2021 (26). BUSCO values represent complete, single copy, duplicated, fragmented, and missing single-copy orthologue genes.

**FIG 1:**
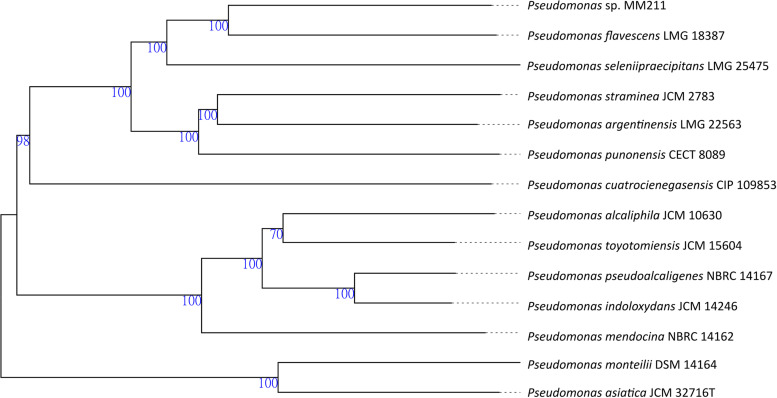
Genome BLAST Distance Phylogeny (GBDP) tree. The phylogenetic tree was created with the Type (Strain) Genome Server (TYGS) (22). The tree was inferred with FastME (v2.1.6.1) (23) from GBDP distances calculated from genome sequences. The branch lengths are scaled in terms of GBDP distance formula *d_5_*. The numbers at the branches are GBDP pseudo-bootstrap support values of >60% from 100 replications, with an average branch support of 100.0%. The tree was rooted at the midpoint (24).

The genome sequence of Pseudomonas sp. strain MM211 presented here has Pseudomonas flavescens LMG 18387 (GenBank accession number GCA_900100535.1) (16) and Pseudomonas seleniipraecipitans LMG 25475 (GenBank accession number GCA_900102335.1) (17) as its closest relatives (Fig. 1). The digital DNA-DNA hybridization (dDDH) shows values of 41.8% with P. flavescens LMG 18387 and 36.4% with P. seleniipraecipitans LMG 25475, both well below the 70% cutoff value for dDDH (18). A carotenoid biosynthetic gene cluster was identified using the antiSMASH server (19, 20). A KEGG analysis showed that Pseudomonas sp. strain MM211 is likely able to grow a flagellum (21). Furthermore, MM211 may be auxotrophic for biotin. P. flavescens, the most closely related species, is also capable of producing a flagellum and pigments (16).

### Data availability.

The MM211 assembly, RefSeq annotation, and reads are available at NCBI GenBank under accession numbers GCA_020386635.1, CP081942.1, and SRR15526917, respectively.
